# Uncovering the Secretion Systems of *Acinetobacter baumannii*: Structures and Functions in Pathogenicity and Antibiotic Resistance

**DOI:** 10.3390/antibiotics12020195

**Published:** 2023-01-17

**Authors:** Pu Li, Sirui Zhang, Jingdan Wang, Mona Mohamed Al-Shamiri, Bei Han, Yanjiong Chen, Shaoshan Han, Lei Han

**Affiliations:** 1 School of Public Health, Xi’an Jiaotong University Health Science Center, Xi’an 710061, China; 2 Department of Microbiology and Immunology, School of Basic Medical Sciences, Xi’an Jiaotong University Health Science Center, Xi’an 710061, China; 3 Department of Hepatobiliary Surgery, The First Affiliated Hospital of Xi’an Jiaotong University, Xi’an 710061, China

**Keywords:** *Acinetobacter baumannii*, secretion systems, pathogenicity, antibiotic resistance

## Abstract

Infections led by *Acinetobacter baumannii* strains are of great concern in healthcare environments due to the strong ability of the bacteria to spread through different apparatuses and develop drug resistance. Severe diseases can be caused by *A. baumannii* in critically ill patients, but its biological process and mechanism are not well understood. Secretion systems have recently been demonstrated to be involved in the pathogenic process, and five types of secretion systems out of the currently known six from Gram-negative bacteria have been found in *A. baumannii*. They can promote the fitness and pathogenesis of the bacteria by releasing a variety of effectors. Additionally, antibiotic resistance is found to be related to some types of secretion systems. In this review, we describe the genetic and structural compositions of the five secretion systems that exist in *Acinetobacter*. In addition, the function and molecular mechanism of each secretion system are summarized to explain how they enable these critical pathogens to overcome eukaryotic hosts and prokaryotic competitors to cause diseases.

## 1. Introduction

*Acinetobacter baumannii* is a strictly aerobic, non-fermenting, Gram-negative coccobacillus with pili and capsule, but no flagella. It is ubiquitous in nature, and used to be considered to be of negligible significance due to its low virulence [1]. However, the rapidly increasing nosocomial infections and high mortality caused by *A. baumannii*, as well as its strong drug resistance, have raised people’s attention [2]. Taken together with *Enterococcus faecium*, *Staphylococcus aureus*, *Klebsiella pneumonia*, *Pseudomonas aeruginosa*, *Enterobacter*, *Acinetobacter baumannii* has been enrolled as a member of ESKAPE by the Infectious Diseases Society of America (IDSA) in order to emphasize the importance of these pathogens in causing hospital infections and resisting the effects of a variety of antimicrobial drugs [3,4].

The high frequency of *A. baumannii* nosocomial infections is closely related to its strong environmental persistence. *A. baumannii* can survive in nutrient-limited and desiccation environments, and is capable of resisting disinfections [5]. Moreover, it is able to survive for long periods of time on both biotic and abiotic surfaces [6]. Based on these advantages, *A. baumannii* can be easily transmitted patient to patient by air, water, and contact with medical personnel’s hands and equipment, thus colonizing multiple sites and finally leading to a variety of infections, such as pneumonia, septicemia, urinary tract infections, meningitis, and skin and wound infections [7,8,9].

Antibiotic resistance is another key factor that contributes to *A. baumannii* infections and outbreaks. The increasing rate of infections caused by drug-resistant *A. baumannii* is a significant issue in hospitals all over the world [10]. The continued overuse and misuse of antibiotics enable *A. baumannii* to develop different types of resistance mechanisms, e.g., the acquisition of multiple antibiotic resistance genes to produce degradative enzymes, a decrease in bacterial membrane permeability, the alteration of antibiotic targets, the overexpression of efflux pumps, a change in metabolic status, and the formation of biofilms [11]. Therefore, this bacterium can escape the killing of antibiotics and conquer the stress conditions, further leading to infections. *A. baumannii* has an extraordinary genetic plasticity that results in a high capacity to acquire antimicrobial resistance traits [2], thus producing many multidrug-resistant (MDR), extensively drug-resistant (XDR), and even pan-drug-resistant (PDR) strains, representing a significant challenge for therapy in clinics.

Infections are also dependent on virulence factors. Various genes have been revealed to be involved in the pathogenic procedures of iron acquisition, nutrient uptake, adhesion, biofilm formation, invasion, hemolytic activity, and cytolytic activity [12,13]. Among them, protein secretion systems have received much attention. They can transport the virulence factors produced by bacteria into extracellular environments, meaning that the latter will manipulate the host’s defenses and facilitate pathogen infection [14,15]. Until recently, six secretion systems from Gram-negative bacteria have been revealed and studied; namely, type I secretion system (T1SS) to type VI secretion system (T6SS). Some of these have been characterized and reported to have specific roles in the pathophysiology of *A. baumannii*, whereas the gene and protein structures of some secretion systems in *A. baumannii* are still not clear and are being explored. Moreover, the association between secretion systems and drug resistance has been discovered in some bacteria, e.g., the T3SS in *Pseudomonas aeruginosa* correlates with a fluoroquinolone resistance phenotype, and the T4SS in many Gram-negative pathogens mediates antibiotic resistance via conjugation [16,17,18]. Meanwhile, the contribution of secretion systems to antibiotic resistance in *A. baumannii* is poorly understood. Here, an overview of the progress of the research on the structure, composition, pathogenicity, and relation to antibiotic resistance of the secretion systems in *A. baumannii* is presented.

## 2. Type I Secretion System (T1SS)

The T1SS is a highly conserved secretion system in pathogenic Gram-negative bacteria. However, it is less reported in *A. baumannii*. In 2017, the T1SS was first identified in the pathogenic *Acinetobacter nosocomialis* strain M2 upon bioinformatic analysis by Harding et al. [19]. Until now, only two reports have described the structure and function of the T1SS in *Acinetobacter* [19,20].

### 2.1. Gene and Structure

**Figure 1:**
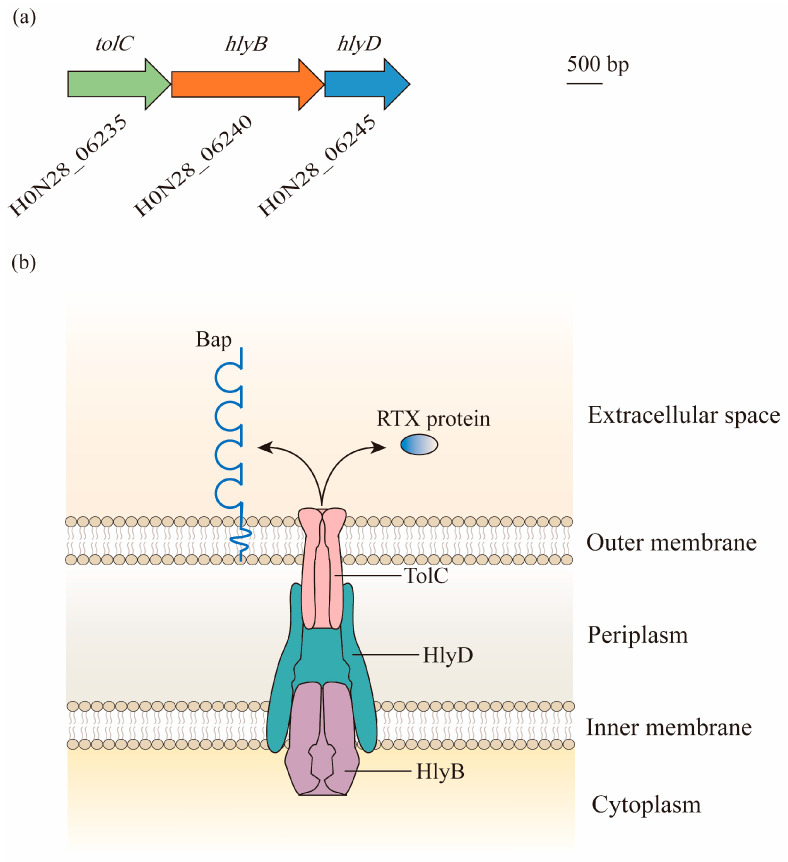
Composition and structure of the type I secretion system (T1SS) in *A. baumannii*: (**a**) Bioinformatic analysis has led to the identification of the T1SS in genomes of *A. baumannii*. Gene locus tags are cited from ATCC 17978. Genes predicted to encode proteins required for the biogenesis of the T1SS are found in three gene clusters, with *hlyB* overlapping with *tolC* and *hlyD*. (**b**) The three components of the T1SS act together to facilitate the secretion of effectors. TolC is a trimeric outer membrane protein with the α-helical barrel forming a tunnel through the periplasm, and it interacts with HlyD. HlyD has a large periplasmic domain linked by a single transmembrane helix, which anchors in the inner membrane. The energy required for the export of specific T1SS substrates is provided by HlyB, which is an ATP-binding protein. Two putative T1SS effectors, namely, Repeats-in-Toxin (RTX)-serralysin-like toxin and biofilm-associated protein (Bap), are involved in the formation and stability of biofilm.

The locus that is homologous to the prototype T1SS of *Escherichia coli* containing the *tolC*, *hlyB*, and *hlyD* genes is found in the M2 chromosome, as well as in *A. baumannii*. In contrast to *E. coli*, these genes are found in three gene clusters, and are most likely in an operon, given that the open reading frame (ORF) for *hlyB* overlaps with both *tolC* and *hlyD* [19] (Figure 1a).

This *tolC*-*hlyB*-*hlyD* gene cluster produces three proteins with high molecular weights of 130 kDa, 250 kDa, and 70 kDa. They form a secretion system with the elements of TolC, which is localized in the outer membrane, HlyB, which is anchored in the inner membrane as an ATP-binding cassette transporter, and HlyD as a periplasmic adaptor [19] (Figure 1b).

### 2.2. Function

#### 2.2.1. Secretion of Putative Effectors

The T1SS facilitates the secretion of two putative effectors from the cytoplasm to the extracellular milieu, including Repeats-in-Toxin (RTX)-serralysin-like toxin and the biofilm-associated protein (Bap) [19]. The former belongs to the RTX family, which is a heterogeneous group of proteins translocated out of Gram-negative bacteria by the T1SS. They are commonly involved in bacterial adhesion, pathogenesis, and biofilm formation [21], but the role that RTX-serralysin-like toxin plays in *Acinetobacter* is not yet entirely understood. By contrast, Bap has been well studied. The Bap protein identified from clinical *A. baumannii* isolates that lead to bloodstream infections is homologous to *Staphylococcus aureus*, with nucleotide sequences consistent with cell surface adhesion molecules. It is one of the largest bacterial proteins that localize on the surface of *A. baumannii* and has a remarkably low isoelectric point (pI) at 2.9, making it one of the most acidic bacterial proteins [22,23]. Bap was found to be necessary for mature biofilm formation on medically relevant surfaces, demonstrating the importance of the three-dimensional tower structure and water channel formation. Moreover, it was also involved in the adherence of *A. baumannii* to eukaryotic cells, including human bronchial epithelial cells and neonatal keratinocytes, which is a key step in the biofilm formation of this bacterium in the host [24]. The absence and mutations of Bap diminished both the biovolume and thickness of the biofilm in *A. baumannii* [23]. Interestingly, a stronger biofilm formation was correlated with the overexpression of Bap under the condition of a low iron concentration [25]. As Bap was secreted via the T1SS, Harding et al. further verified that the *Acinetobacter* T1SS was required for biofilm formation [19]. Additionally, the acidic protein Bap may influence the physical and chemical properties of a variety of antibiotics, thus resulting in drug resistance [26].

#### 2.2.2. Cross-Talk with other Secretion Systems

The T1SS has also been revealed to cross-talk with other secretion systems [19]. For example, compared with the wild-type *Acinetobacter* strain, several T2SS-associated proteins, such as CpaA, LipH, a rhombosortase, and a rhombotarget, were found in lower quantities in the T1SS mutant. Moreover, the activity of the T6SS in minimal medium was repressed by the deletion of the T1SS system. This was due to the significantly lower level of the T6SS-associated proteins, VgrG and Hcp, in the T1SS mutant. Specifically, mutations in any component of the T1SS reduced Hcp secretion under nutrient-limited conditions, whereas that in PilD, which is a prepilin peptidase necessary for both T4P and T2SS systems, did not alter Hcp secretion, suggesting a specific association between the T1SS and T6SS. Lastly, two distinct functioning contact-dependent inhibition (CDI) systems were found in pathogenic *A. baumannii* strains. CDI systems are independent from the T1SS and T6SS that facilitate inhibition of the growth of neighboring bacteria and are found to be conserved in medically relevant *Acinetobacter*. However, in the T1SS mutant, a predicted CDI-associated protein was identified at a significantly lower level, indicating the cross-talk between them.

#### 2.2.3. Virulence

The virulence of *A. baumannii* is associated with the T1SS. As observed by Harding et al., T1SS mutants showed attenuated virulence in a *Galleria mellonella* infection model [19]. Moreover, in a recent work, Sycz et al. [20] reported a clinical urinary *A. baumannii* isolate, UPAB1, which was able to replicate in macrophages and escape from them by lysing the host cells. The T1SS was demonstrated to be required for UPAB1 in the process of intracellular replication by secreting two common T1SS-dependent effectors, BapA and RTX2, as well as some additional effectors including proteases, phosphatases, glycosidases, and a putative invasion. Interestingly, the orthologs of this invasion from other bacteria were required to induce bacterial entry and to suppress reactive oxygen species (ROS) generation by the host macrophages [20,27].

## 3. Type II Secretion System (T2SS)

The T2SS is a multiprotein secretion system that is widely distributed in Gram-negative bacteria, including enterotoxigenic *Escherichia coli*, *Legionella pneumophila*, *Vibrio cholerae*, *Pseudomonas aeruginosa*, and *Klebsiella pneumoniae* [28,29,30,31,32,33,34]. It was first reported in *A. baumannii* in 2014 and was subsequently shown to be active in ATCC 17978 by Johnson et al. [35,36]. Further, the T2SS is found in the majority of *A. baumannii* genomes.

### 3.1. Gene and Structure

**Figure 2:**
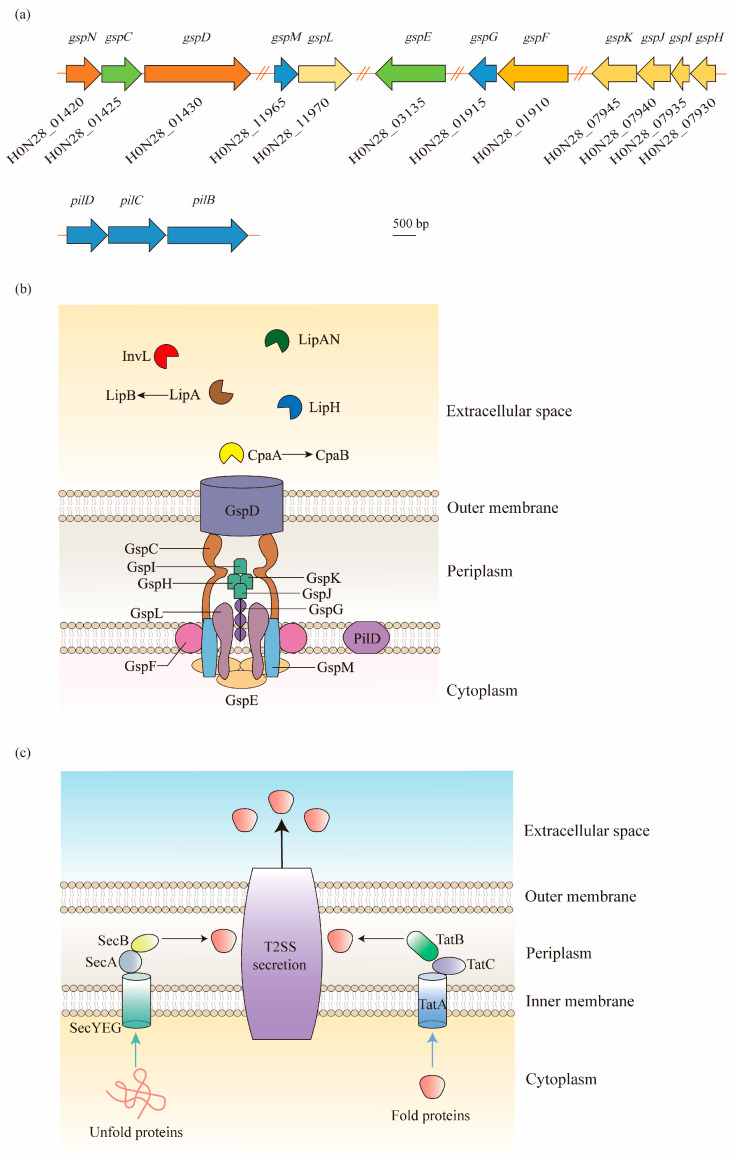
Type II secretion system (T2SS) structure of *A. baumannii* and its protein secretion mechanism: (**a**) As shown in the ATCC 17978 genome, the *gsp* genes required for the T2SS are located in five distant loci, and a single prepilin/pre-pseudopilin peptidase homolog is located in the *pilBCD* cluster. (**b**) The T2SS is composed of an outer membrane (OM) complex (GspD), a periplasmic pseudopilus (GspG, GspH, GspI, GspJ, and GspK), and an inner membrane (IM) platform (GspC, GspF, GspL, and GspM), which relates to the cytoplasmic ATPase GspE. In *A. baumannii*, the T2SS shares a processing protein, PilD, with type IV pili. The T2SS secretes a large number of effectors required for virulence, including the metallopeptidase CpaA (chaperone CpaB), the lipoyl synthases LipA (chaperone LipB), LipH, and LipAN, and a novel lipoprotein, InvL. (**c**) The T2SS-dependent proteins are first exported across the IM to the periplasm via the Sec or Tat pathways in *A. baumannii*. The Sec pathway primarily translocates unfolded proteins, relying on a hydrophobic signal sequence at the N-terminus. On the contrary, the Tat pathway, consisting of TatA, TatB, and TatC, primarily secretes folded proteins. Afterwards, the signal sequence is cleaved, followed by the folding of proteins. Finally, the folded proteins are expelled extracellularly through the OM channel.

In *Acinetobacter* spp., the T2SS is encoded by 12 essential genes, namely, *gspC-M* and *pilD*, and forms an apparatus spanning both the inner membrane and outer membrane [37,38] (Figure 2). In contrast to other Gram-negative pathogens, the core *gsp* genes are not organized in one or two operons, but are grouped into five distinct gene clusters scattered throughout the *Acinetobacter* genome [39] (Figure 2a).

In general, the T2SS consists of four parts: (1) an outer membrane (OM) complex; (2) a periplasmic pseudopilus; (3) an inner membrane (IM) complex called the assembly platform (AP); and (4) a cytoplasmic ATPase [40]. The OM complex is composed of GspD, which forms a secretin channel across the outer membrane to transport substrates from the periplasm to the extracellular milieu [41]. The IM platform is composed of GspC, GspF, GspL, and GspM, in which GspC is joined to the periplasmic domains of GspD, thereby connecting the IM platform with the OM complex. In between the OM and IM complexes, the periplasmic pseudopilus, a structure homologous to the type IV pilus, is attached to the IM platform with the composition of major pseudopilin GspG and minor pseudopilins GspH, GspI, GspJ, and GspK. Before the assembly of these subunits, PilD is involved in the cleavage and methylation procedure. Additionally, the cytoplasmic ATPase is formed by a hexamer protein, GspE, to provide ATP to the T2SS for the secretion of effector proteins [40,42] (Figure 2b).

### 3.2. Function

#### 3.2.1. Secretion of Enzymes and Toxins

The T2SS is an important virulence factor that can secrete multiple degradative enzymes and toxins that associate with the fitness of *A. baumannii* in various conditions, including the external environment and mammalian host [43]. The secretion by the T2SS undergoes a two-step process (Figure 2c). Firstly, the substrates of the T2SS, commonly possessing an N-terminal signal peptide, are translocated from the cytoplasm to the periplasm via the general export (Sec) pathway or twin arginine translocation (Tat) pathway. Secondly, after the cleavage of the signal sequence, the proteins fold into a tertiary and/or quaternary structure and exit the bacterial cell through the OM channel [34,43]. After investigation through bioinformatics, proteomics, and mutational analyses, *Acinetobacter* was found to export several substrates through the T2SS, including lipases LipA, LipH, and LipAN, as well as the protease CpaA and lipoprotein InvL [39,44,45,46].

LipA, after being transferred extracellularly, was reported to be required by *A. baumannii* for utilizing exogenous lipids to obtain nutrients and benefit for colonization in a murine bacteremia model [36]. Its secretion and activity are also related to the chaperone protein LipB, except in the T2SS [34]. Similar to LipA, the secretion of LipH is dependent on a functional T2SS. LipH was discovered to mediate lipase activity as there was residual lipase activity of the culture supernatants in the absence of LipA. Additionally, LipH was found to be secreted by not only the *A. nosocomialis* strain M2, but also a panel of *Acinetobacter clinical strains*, *including A. baumannii* [39]. LipAN is a newly discovered T2SS-dependent phospholipase from *A. baumannii* ATCC 17978, found in 2016. It locates on the plasmid and contributes to the lung colonization of *A. baumannii*, as investigated in a mouse pneumonia model [44].

CpaA, a zinc-dependent metallo-endopeptidase, was first purified from the culture supernatant of an *A. baumannii* clinical isolate in 2014. It is conserved in most clinical isolates of *A. baumannii*, but it does not exist in ATCC 17978 or ATCC 19606 [45]. Along with LipA and LipH, the potential virulence factor CpaA is also secreted by the T2SS [39]. CpaA is composed of four glycan-binding domains that facilitate this protease to display glycoprotein-targeting activity [47]. It can cleave two *O*-linked glycoproteins, factors V and XII, finally leading to the deregulation of the human blood coagulation system [45,48]. In a recent work, more *O*-linked human glycoproteins were shown that could be cleaved by CpaA, such as CD55 and CD46, that are involved in complement activation, and its activity is unaffected by sialic acid [49]. Similarly to LipA, the chaperone CpaB is essential for the stability and secretion of CpaA. CpaB is a membrane-bound T2SS chaperone that strongly interacts with CpaA in a CpaAB complex with the stoichiometry of 1:1, where the protease (CpaA) surrounds the chaperone (CpaB). However, the proteolytic activity of CpaA is not blocked by the binding of CpaB. This complex structure is a novel model for chaperone–protease interaction [47].

A newly discovered lipoprotein, InvL, is identified as the first effector of the T2SS belonging to the intimin-invasion family. InvL was primarily found in the insoluble fractions of the supernatant of a urinary isolate, UPAB1, and further revealed to be surface-localized. InvL is found to be closely related to international clone (IC) 2 [46]. Its secretion and surface exposure were found to be dependent on the T2SS, since InvL-His_6_ expressed in the *gspD*^+^ strain was readily degraded by proteinase K, whereas the degradation in the Δ*gspD* mutant failed [46].

#### 3.2.2. Pathogenesis

The T2SS plays an important role in infections of *A. baumannii*, primarily by secreting numerous effector substrates, such as the above-mentioned lipases and proteases, to the cell surface or extracellular space. These proteins act on the external environments or target cells, and contribute to the acquisition of nutrients, thus maintaining the survival and colonization of bacteria in the host [50]. Johnson et al. [36] revealed that *A. baumannii* ATCC 17978 mutants lacking either T2SS components GspD and GspE, or its secretion substrate LipA, were unable to grow in vitro when long-chain fatty acids were supplemented as the sole source of carbon. A negative impact on the in vivo fitness of these mutants was also observed in immune-deficient mice.

Moreover, T2SS contributes to the pathogenesis of *A. baumannii* through LipAN, which is a phosphatidylcholine-degrading phospholipase C that displays phospholipase activity and benefits for the improved colonization of *A. baumannii* in the lungs of infected mice [44]. Indeed, by comparing the secretome of *A. baumannii* ATCC 17978 with that of the highly virulent MDR strain 5075, Elhosseiny et al. verified that the T2SS and its secretion substrates provided a colonization advantage to *A. baumannii* 5075 over ATCC 1797, but was more important to the latter for biofilm formation [51]. Similarly, the T2SS-dependent protease CpaA is necessary for the dissemination of *A. nosocomialis* from the initial infection site in the lungs to a distal site in the spleen [52]. The invasion-like adhesin InvL is capable of binding to extracellular matrix (ECM) components, in which fibrinogen shows the highest affinity with it, thus mediating the adhesion to urinary tract cells. Moreover, the *invL* mutant is attenuated in the catheter-associated urinary tract infection (CAUTI) model, verifying that the T2SS plays an important role in the uropathogenesis of *A. baumannii* through InvL [46].

Furthermore, the T2SS and its substrates also participate in the immune escape effect. Waack et al. [43] noticed that the loss of *gspD* resulted in a remarkable reduction in bacterial survival in human serum lacking factor C1q, which is a component of the classical complement pathway; however, it had no such effect in the absence of factor B, which mediates the alternative complement pathway. These findings indicate that the T2SS mediates the outer membrane translocation of an effector protein, thus contributing to in vivo fitness by protecting *A. baumannii* from the human alternative complement pathway [43].

In conclusion, *A. baumannii* mediates the release of various virulent substances through the T2SS and facilitates the adaptation of this organism to the environments and hosts, thus enhancing its ability to cause diseases.

#### 3.2.3. Antibiotic Resistance

Drug resistance is a less mentioned topic in the T2SS field. However, Elhosseiny et al. [51] recently discovered that the T2SS was involved in the resistance of the fluoroquinolone antibiotic ciprofloxacin in AB5075, where an eight-fold increase in the MIC value of ciprofloxacin was detected in the *gspD* loss mutant, and the value was restored upon mutant complementation. The altered expression of outer membrane porins or efflux pumps that are controlled by the T2SS may contribute to antibiotic resistance; however, further research is required to confirm this speculation.

## 4. Type IV Secretion System (T4SS)

T4SSs are multiprotein nanomachines, widespread in Gram-negative and Gram-positive bacteria, that deliver macromolecules, e.g., DNA and protein, to bacterial recipients or eukaryotic target cells [53]. They are generally divided into three groups; namely, type F and P (IVA), IVB, and GI systems [54,55]. However, T4SSs are less reported in *A. baumannii*. The information can be summarized from five studies, as discussed below. By using the high-density pyrosequencing method, the elements homologous to the Legionella/Coxiella T4S apparatus were first discovered in *A. baumannii* ATCC17978 [56]. Later, in a pathogenic isolate, ACICU, the plasmid pACICU2 was found harboring a complete *tra* locus, which encoded the conjugative apparatus and an F-type T4SS (based on the F-plasmid of *Escherichia coli*) [57]. However, the structure and function of the *A. baumannii* T4SS were not illustrated in these two studies. Furthermore, the plasmid replicase (*rep*) gene *rep*Aci6 from pACICU2 was found widely distributed in *A. baumannii* clinical strains, which carried the T4SS protein TraC coding gene [58,59]. Thus, *rep*Aci6 served as a candidate for screening the F-type T4SS, and the plasmid carried the genes required for the biogenesis of the T4SS, such as *traC*, *traD*, and *traU*, which were identified in clinical carbapenem-resistant *A. baumannii* (CRAB) isolates [60].

### 4.1. Gene and Structure

**Figure 3:**
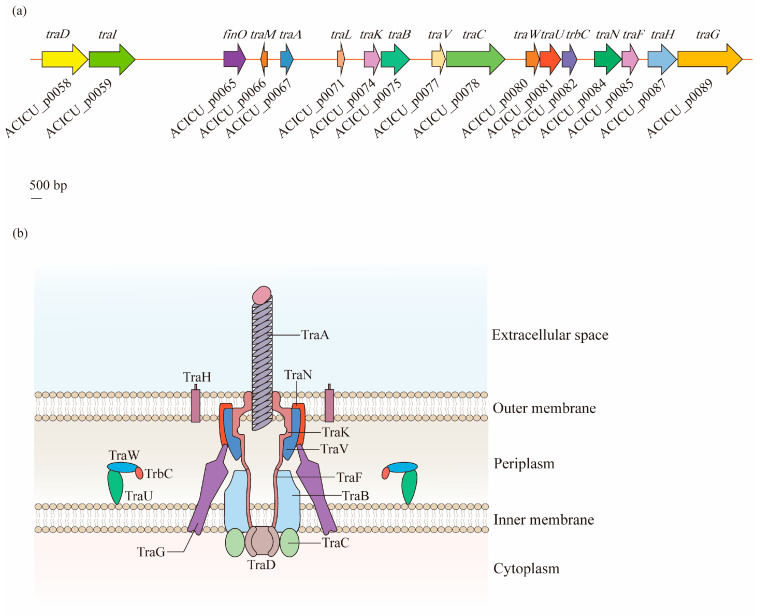
Structural organization of the type IV secretion system (T4SS) in *A. baumannii*: (**a**) Discovered in the *A. baumannii* ACICU plasmid pACICU2, the F-type T4SS contains a series of *tra* operon genes, and two other genes, *trbC* and *finO*. (**b**) The T4SS is a highly sophisticated nanomachine spanning the entire bacterial cell envelope in *A. baumannii*. The F-like T4SS apparatus is composed of a pilus assembly component (TraA), a core complex (TraK, TraV, TraN, and TraH) embedded in the outer membrane (OM), an inner membrane (IM) platform (TraF, TraB, TraG, TraU, TraW, and TrbC), and components of the cytoplasm (TraC and TraD).

The F-type T4SS in *A. baumannii* contains a series of *tra* operon genes, including *traA*, *traB*, *traC*, *traD*, *traE*, *traF*, *traG*, *traH*, *traI*, *traK*, *traL*, *traM*, *traN*, *traU*, *traV*, and *traW*, as well as another two genes, *trbC* and *finO*. Through the alignment of seven F-like *A. baumannii* plasmids, it was observed that the core genes involved in pilus biosynthesis (*traA*, *traB*, *traC*, *traF*, *traH*, *traK*, *traU*, *traV*, *traW*, and *trbC*), nicking (*traI*), the initiation of transfer (*traM* and *traD*), mating aggregate stabilization (*traN* and *traG*), and regulation (*finO*) were highly conserved [60] (Figure 3a).

According to the analysis of Liu et al. [60], the T4SS of *A. baumannii* is a symmetrical barrel-shaped structure that is divided into the following units: (1) the pilus assembly component localized in the extracellular space across the OM (TraA); (2) the core complex embedded in the OM (TraK, TraV, TraN, and TraH); (3) the constituents of an IM platform (TraF, TraB, TraG, TraU, TraW, and TrbC); and (4) the components of the cytoplasm (TraC and TraD). This structure is similar to that of the typical VirB/D4 T4SS, which exists on the *Agrobacterium tumefaciens* Ti plasmid, and has gene consistency with *tra* operons as *traB*/*virB10*, *traC*/*virB4*, and *traD*/*virD4* [53,61] (Figure 3b).

### 4.2. Function

Although there has not been any empirical evidence demonstrating the function of the T4SS in *A. baumannii*, it is rational to speculate that it has similar performances to the universal T4SSs in other bacteria, as they share similar structures [60,62].

#### 4.2.1. DNA Exchange and Antibiotic Resistance

Generally speaking, T4SSs are ancestrally related to bacterial conjugation machines, which mediate the transfer of genes and proteins across membranes [61]. T4SSs can recognize DNA substrates and translocate them to recipient bacterial cells by conjugative transfer. In this way, horizontal gene transfer is performed to disseminate mobile genetic elements, such as antibiotic resistance genes, virulence genes, and other fitness traits, to benefit bacteria by enhancing their survival in various environments and promoting the evolution of infectious pathogens [61]. The spreading of antibiotic resistance genes will lead to the rapid development of drug-resistant bacteria and even cause outbreaks of nosocomial infections. The conservation of most T4SS genes between *A. baumannii* and *E. coli* K-12 indicates that the function of the T4SS could be essential and unique in conjugation-mediated gene transfer [60].

Moreover, instead of connecting with the donor cells, T4SSs can either release naked DNAs to the milieu, or take up DNAs from the extracellular environments, therefore fulfilling the exchange of DNAs with the milieu [62,63].

#### 4.2.2. Virulence

T4SSs can also act as effector translocator systems that deliver bacterial effector proteins across both the membrane of bacteria and eukaryotic target cells, finally contributing to bacterial pathogenicity by assisting the colonization and propagation of bacteria in the eukaryotic host, as well as the activation of pro-inflammation, apoptosis, and cytoskeleton rearrangements of host cells [54,64,65]. In addition, T4SSs are able to deliver a killing toxin to the bacterial neighbors to maintain the advantage of survival [62]. The T4SS may, to a large extent, contribute to the pathogenesis of *A. baumannii*. However, further research needs to be performed to investigate its exact functions.

## 5. Type V Secretion System (T5SS)

The T5SS, also known as the autotransporter, is a series of simple protein export pathways that are distributed in a large range of Gram-negative bacteria [66]. They are classified into monomeric autotransporters (MA), trimeric autotransporters (TA), and two-partner secretion systems (TPSS), with the composition of a single polypeptide for MA and TA, and separate polypeptide chains for TPSS [67,68]. Depending on the different structural features and domain organization, the T5SS is divided into five known subclasses, so-called types Va to Ve, and possibly another recently identified type, Vf [68]. However, only two types, Vb and Vc, have been identified in *A. baumannii* [34]. Therefore, type Vb and type Vc will be the focused of this review.

### 5.1. Gene and Structure

**Figure 4:**
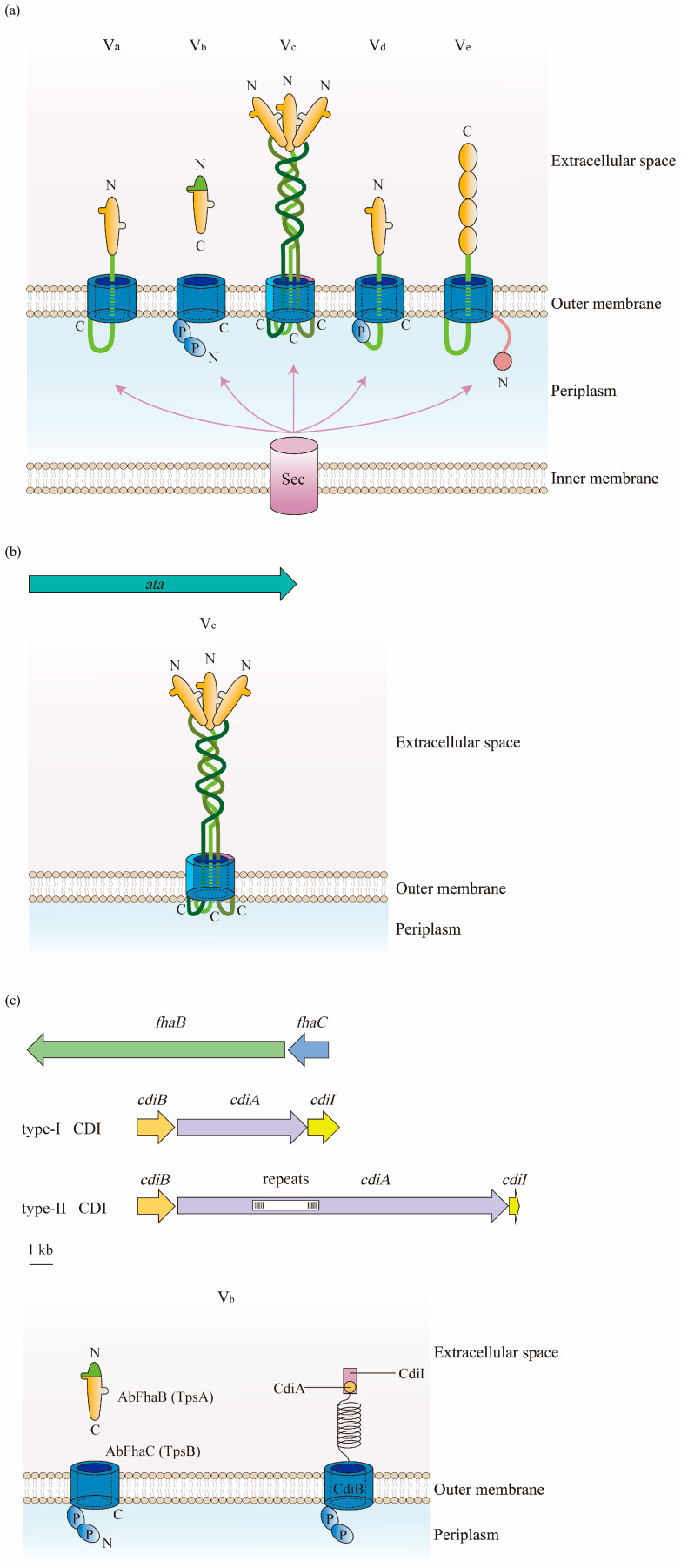
Structure of the type V secretion system (T5SS) in *A. baumannii*: (**a**) There are five types of T5SS in Gram-negative bacteria. They consist of three parts: a signal sequence at the N-terminus, a secreted passenger in the extracellular milieu, and a transporter at the C-terminal. β-Barrels are displayed in blue; linkers and the two-partner secretion (TPS) domains are in green; passenger regions are in orange; polypeptide transport-associated (POTRA) domains are labeled as P; and the N- and C-termini are indicated. The translocation of substrates for subclasses of T5SS from the cytoplasm to the periplasm relies on the Sec pathway. (**b**) Type Vc is the most frequently identified T5SS in *A. baumannii*. It is formed by a trimeric protein, Ata, which contains a signal peptide at the N-terminus, a surface-exposed passenger domain, and a C-terminal domain. (**c**) Two forms of type Vb are found in *A. baumannii*. The one belonging to the TPSS is constructed of AbFhaB and AbFhaC, which represent TpsA and TpsB in other Gram-negative bacteria, respectively. AbFhaB (TpsA) is the passenger domain that is secreted out of cells through the outer membrane (OM) by AbFhaC (TpsB), which is the translocator domain located in the OM. Another one is the contact-dependent inhibition (CDI) system composed of CdiA and CdiB. Similar to TpsA, the toxin CdiA is released from the periplasm to the cell surface by the OM transporter CdiB.

In contrast to other types of secretion systems that span the entire cell envelope with a syringe-shape structure, the T5SS only spans the OM. The T5SS consists of three major regions; namely, a signal sequence at the N-terminus, an extracellular secreted passenger, and a β-barrel domain (transporter) at the C-terminal that anchors the protein to the bacterial OM [68,69] (Figure 4a). Being produced in the cytoplasm, the protein is recognized at the N-terminal signal peptide, which targets the Sex complex to mediate the inner-membrane translocation of the protein to the periplasm [34]. Thereafter, the C-terminal transporter domain inserts into the OM and secretes the protein to the external environment through its OM pore. Finally, the passenger domain located between the signal peptide and the β-barrel domain displays the specific effector function extracellularly after proteolytic cleavage [40].

Type Vc is the most popular T5SS in the *A. baumannii* chromosome that belongs to the TA family. Therefore, the protein of type Vc in this bacterium is designated as the *Acinetobacter* trimeric autotransporter (Ata) [70]. Encoded by the *ata* gene, the autotransporter Ata contains a long signal peptide followed by an N-terminus, a surface-exposed passenger domain, and a C-terminal domain encoding four β-strands [70] (Figure 4b).

In contrast to classical autotransporters, type Vb belongs to TPSS, where the passenger and translocator (β-barrel) domains locate in two distinct polypeptide chains that are formed by TpsA and TpsB [67]. TpsA and TpsB are encoded in one operon, and the former connects at the polypeptide transport-associated (POTRA) domain of the latter for secretion through the OM to either be surface-displayed or transported extracellularly [71]. In this way, when releasing the passenger out of the cells after being transported by the β-barrel domain, there is no need for release by proteolytic cleavage [68]. In the *A. baumannii* strain AbH12O-A2, AbFhaB and AbFhaC were found to represent TpsA and TpsB, respectively, due to the highly conserved structure of these proteins [72] (Figure 4c).

Another type of Vb recently observed in *A. baumannii* is the CDI system composed of CdiA and CdiB. CdiA is a large multi-domain protein that forms a filament folded as a β-helix, similarly to TpsA, and has a C-terminal toxin domain. The CdiA protein in the periplasm is released to the cell surface by the OM transporter CdiB, and its β-helix presents the toxin domain to the neighboring bacteria, finally inhibiting their growth [73]. A cytoplasmic immunity protein, CdiI, is also expressed by the CDI operon to protect bacteria from fratricide and auto-inhibition by CdiA toxins [74,75,76] (Figure 4c).

### 5.2. Function

T5SSs play crucial roles in the virulence of Gram-negative bacteria. The function of the passenger domain from different bacteria is highly diverse, where it can be enzymatic, proteolytic, toxic, or adhesive, thus contributing to bacterial virulence in colonization, intracellular mobility, nutrient acquisition, immune evasion, the alteration of host cell processes, and biofilm formation [68,77].

#### 5.2.1. Function of Type Vc System

In *A. baumannii*, the type Vc autotransporter, Ata, is present in many clinical isolates, and is reported to be produced at the highest level during the very early exponential phase. Ata is critical for biofilm formation, binding to various extracellular/basal matrix proteins, including collagen types I, III, IV, and V and laminin. The biofilm formed on adherent cells was significantly lower in the *ata* deletion mutant. In addition, Ata mediates the virulence of *A. baumannii* by binding to collagen type IV, which promoted the survival of strains in a mouse model of lethal infection [70]. Further study revealed that Ata bound to host glycans with high affinity, including galactose, N-acetylglucosamine, and galactose (β1-3/4) N-acetylglucosamine. This ability was crucial for Ata to recognize human plasma fibronectin during host adherence, as deglycosylated fibronectin had no interaction with Ata [78]. In addition to adhesion, Ata also mediates the in vivo invasion of *A. baumannii* and induces the apoptosis of the host cells [79].

#### 5.2.2. Function of Type Vb Systems

The type Vb systems, including AbFhaB/FhaC and CdiA/CdiB, also play potential roles in the pathobiology of *A. baumannii*. The AbFhaB/FhaC system is involved in the attachment and fibronectin-mediated adherence to host cells. Moreover, these systems participate in the virulence of *A. baumannii*, where higher fertility and survival rates were monitored in *Caenorhabditis elegans* and mouse infection models, respectively, when *fhaC* was absent [72]. In contrast, the CDI system is reported to inhibit the growth of non-immune neighboring cells and, on the other hand, to favor the formation of biofilm structures, thus promoting social interactions between CDI^+^ cells to facilitate biofilm formation [80].

## 6. Type VI Secretion System (T6SS)

The T6SS is a multiprotein transmembrane nanomachine discovered in numerous Gram-negative bacteria, including *Vibrio cholerae*, *Escherichia coli*, *Pseudomonas aeruginosa*, *Klebsiella pneumoniae*, *Francisella tularensis*, and *Yersinia pseudotuberculosis* [81]. It is syringe-shaped and is commonly used by bacteria to inject toxic effectors into competitors or host cells [82]. Several parts of this secretion system are structurally and functionally homologous to the T4 bacteriophage tail, suggesting a common evolutionary origin of this apparatus [83]. In recent years, an increasing number of studies have reported various aspects of the T6SS from *A. baumannii*, including its composition, structure, regulation, and function, confirming it as an important virulence factor.

### 6.1. Gene and Structure

**Figure 5:**
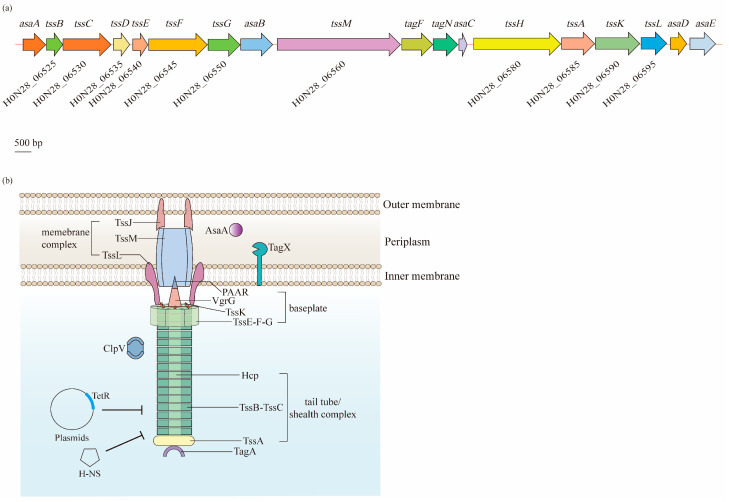
Biogenesis and regulation of the type VI secretion system (T6SS) in *A. baumannii*. The T6SS is a class of macromolecular secretion machines, which translocate proteins into a variety of recipient cells: (**a**) A single gene cluster carries 18 putative genes that are predicted to encode components of the T6SS. Among them, 12 core genes (*tss*) are coded on the chromosome of *A. baumannii* ATCC 17978. (**b**) The T6SS is composed of three main parts: a membrane complex (TssJ, TssL, and TssM), a cytoplasmic baseplate (TssK, TssF, TssG, TssE, VgrG, and PAAR), and a contractile tail tube/sheath complex (Hcp, TssB, TssC, and TssA). The expression of the T6SS is negatively regulated by the TetR-like proteins encoded on the large, conjugative plasmid pAB3 and proteins within the H-NS family.

The T6SS in *A. baumannii* is found in a cluster located in the genome that contains 18 genes, arranged as *asaA*-*tssBC*-*hcp*(*tssD*)-*tssEFG*-*asaB*-*tssM*-*tagFN*-*asaC*-*tssHAKL*-*asaDE*, while genes of *vgrG*, also known as *tssI*, which are scattered in various numbers throughout the genome [84,85]. In these genes, 12 encode the core T6SS proteins (Tss, Hcp, and VgrG), two encode the TagF and TagN that are associated with the T6SS in other bacteria, and five encode the Asa proteins that only appear in *Acinetobacter* spp. [84] (Figure 5a). Based on the Tss core proteins, the T6SS is composed of three main parts: a membrane complex, a cytoplasmic baseplate, and a contractile tail tube/sheath complex (Figure 5b).

Normally, in a wide range of bacteria, the membrane complex consists of the TssJ, TssL, and TssM proteins that span the cell envelope, with the complex anchored in the IM and the tip embedded in the OM, but not crossing it [86]. Notably, TssJ, an OM lipoprotein interacting with TssM, is absent in *A. baumannii* [85]. TssM and TssL have strong homology with the T4bSS proteins IcmF and IcmH (or DotU), respectively [87,88]. TssM is a core component of the T6SS that anchors to the IM through three transmembrane segments [88]. Similarly, the cytoplasmic protein TssL is also bound to the IM, but through a single transmembrane helix. Two residues of TssL in *A. baumannii*, Asp98 and Glu99, are strongly conserved among T6SS-encoding Gram-negative bacteria, and remarkably impact the dynamics, expression, and functionality of this protein [89]. TssM and TssL are involved in the recruitment and secretion of Hcp, and are important for the activity of the T6SS [90].

The baseplate complex is a central piece of the T6SS machinery that consists of six (TssK)_6_-(TssF)_2_-(TssG)_1_-(TssE)_1_ wedges around a central (VgrG)_3_-PAAR spike. It connects the tail to the membrane complex and initiates the polymerization of the tail tube/sheath complex [91]. TssG is the core component of a baseplate wedge, where its C-terminal domain acts as an adaptor to interact with both TssF and TssK. VgrG, which binds to the PAAR-repeat protein at its distal extremity, is essential for the assembly of the Hcp tube, thus significantly contributing to the structure of the T6SS in various bacteria, including *A. baumannii* [92,93,94].

The tail tube/sheath complex is a contractile structure formed by the Hcp tube, TssBC sheath, and TssA cap. Although VgrG locates in the center of the baseplate complex, it is identified as an extension of the Hcp tube, as the central density of the latter is uniform from the first ring docked on top of the (VgrG)_3_-PAAR spike [93]. Normally, the inner Hcp tube assembles onto the base of VgrG and extends into the cytoplasm. Simultaneously, the TssBC helical sheath polymerizes around the Hcp tube in an extended, high-energy “primed” conformation [95]. Additionally, its proximal ring has been suggested to interact with the TssK-TssF-TssG complex [92]. After contraction, the sheath is disassembled by the AAA^+^ ATPase ClpV for a new assembly cycle of an extended sheath [96]. Lastly, TssA is involved in the assembly of Hcp-TssBC, and caps the distal end of this structure [95,97].

In addition to the core components, additional auxiliaries are required for the *A. baumannii* T6SS to ensure the correct assembly and full activity. For example, TagF and TagN were identified to negatively regulate the activity of the T6SS, where the absence of these two proteins increased the secretion of Hcp [98]. Moreover, AsaA was demonstrated to localize in the periplasmic space and affect the assembly or stability of the T6SS by interacting with TssM [99]. Additionally, a novel peptidoglycan hydrolase, TagX, was proposed to be required for the transit of the T6SS machinery across the peptidoglycan layer, thus finally allowing the assembly of the T6SS [98].

### 6.2. Function

#### 6.2.1. Virulence

Similarly to other Gram-negative bacteria, the T6SS is a multifunctional apparatus in *A. baumannii*. Bacterial virulence is the most concerning effect raised by the T6SS. First of all, outcompeting other bacterial competitors is one critical factor to evaluate the virulence. As observed by Kim et al., clinical *A. baumannii* isolates causing bacteremia were shown to outcompete *E. coli* in a T6SS-dependent manner [100]. This is primarily due to the injection of toxic effectors into target cells, which may have bacteriostatic or bactericidal activities [84]. The T6SS effectors that have been characterized to date include NAD(P)^+^ glycohydrolase, ADP-ribosylating toxins, (p)ppApp synthetase, and Rhs [101,102,103,104]. Current research has found a synergistic effect of D-lysine on the peptidoglycanase activity of the T6SS effector Tse4 in *A. baumannii*. Additionally, the T6SS-mediated killing effect on Gram-positive bacteria was also seen with the lethal combination of D-lysine and Tse4 [105]. Moreover, the significantly enhanced efficacy of the T6SS can be induced by toxins that not only kill, but also quickly lyse, competitor bacteria [82]. The injection procedure was revealed to be reliant upon TssB, Hcp, and TssM [84,106].

Secondly, the T6SS is involved in pathogenicity in eukaryotic hosts. Repizo et al. reported that the T6SS was required for the host colonization of *A. baumannii* in the *G. mellonella* model [106]. Furthermore, T6SS-active clinical strains were found to survive better in the presence of human serum and were more frequently detected in patients with a catheter-related bloodstream infection, hematopoietic stem cell transplantation, and immunosuppressive agent therapy [100]. Higher *hcp* expression was found in invasive *A. baumannii* isolates under the status of respiratory infection and could be triggered by the acid environment [107]. VgrG is also involved in the virulence of *A. baumannii*. It was found to be beneficial to cellular adherence and systemic infection in hosts. The number of *vgrG* deletion mutants adhering to human lung epithelial cells was much lower than that of the wild-type strain, and the lethal ability of this mutant to mice was also decreased [108].

#### 6.2.2. Antibiotic Resistance

Antibiotic resistance is also found to be closely related to the T6SS. Dong et al. revealed that the presence of the T6SS in XDR *A. baumannii* isolates was significantly higher than that in MDR strains, followed by that in sensitive isolates [109]. Compared with the wild-type *A. baumannii* strain, an increased resistance to ampicillin/sulbactam and a decreased resistance to chloramphenicol were detected in a *vgrG*-lacking mutant [108]. The T6SS may be a contributor to inter-species horizontal gene transfer (HGT), which is one of the critical drug resistance mechanisms in bacteria. It was revealed to promote HGT by lysing the neighboring *E. coli* cells, whose genes were subsequently gained by *Acinetobacter* [110]. Indeed, when *hcp* was missing from T6SS+ *A. baumannii* A152, the ability of this strain to acquire antimicrobial resistance plasmids from *E. coli* was reduced remarkably [109]. Nevertheless, the function of the T6SS in *A. baumannii* is instead repressed during the intra-species dissemination of multidrug-resistant plasmids. As discovered by Weber et al., the T6SS was negatively regulated by a large resistance plasmid containing TetR-like regulators, which presented in a wide range of *A. baumannii* [111]. Additionally, the dissemination and conjugation of MDR plasmids among *A. baumannii* isolates relied on their distinctive ability to repress the function of the T6SS [112].

## 7. Conclusions and Future Perspectives

Although six types of secretion systems have been well characterized in numerous Gram-negative bacteria over the past decades, the study in this field is in the initial stage for *A. baumannii*. This bacterium is commonly regarded as a low-grade pathogen, whereas, based on the current knowledge, five types of secretion systems have been discovered in this species, including the T1SS, T2SS, T4SS, T5SS, and T6SS. Among them, the T2SS, T6SS, and Ata from the T5SS are the most frequently reported secretion systems in *A. baumannii*, and can coexist in the majority of isolates. They share high similarity with the composition and structure of the secretion systems in other bacteria and are involved in different aspects, such as pathogenicity and antibiotic resistance.

Virulence, mediated by this mechanism, is mainly achieved via the secretion of various effectors, which not only promote the fitness of *A. baumannii* in different environments, but also alter the physiology of its hosts. Further research to investigate additional effectors, as well as their enzymatic activity, will provide more information on the contribution of secretion systems during human infections.

Secretion systems are normally considered to be involved in bacterial virulence, but less is known about their relationship with antibiotic resistance. A few studies have begun to reveal the changes in antibiotic susceptibility in the mutants of some types of secretion systems, such as the T2SS and T6SS in *A. baumannii*. However, the mechanism for drug resistance mediated by secretion systems is not fully understood. Additional work is necessary to confirm the role that various secretion systems play in the antibiotic resistance in *A. baumannii*. Additionally, more functional components and effectors should be genetically investigated to facilitate a deeper understanding of the mechanism.

Treatment and prevention strategies based on secretion systems can be considered. Currently, sulbactam, carbapenems, aminoglycosides, polymyxins, tigecycline, and tetracycline are recommended for the therapy of *Acinetobacter* infections [113]. Among them, carbapenems are known as the “last line of defense” against Gram-negative bacteria and have been the preferred remedy choice for MDR *A. baumannii* infections in the past few decades. However, an increased incidence of resistance towards this sort of antibiotic has been reported in recent years, and carbapenem-resistant *A. baumannii* (CRAB) has become a global threat to human health, as it is commonly associated with a broad range of co-resistance to other antibiotic classes [114,115,116]. Consequently, the World Health Organization (WHO) lists CRAB in the critical group for the research and development of new antibiotics [115]. In addition, its resistance to other antimicrobials also continues to increase. Therefore, new drugs are in urgent need, and those that target secretion systems could be a good choice.

The T2SS and T6SS are the most frequently discovered secretion systems in *A. baumannii*, demonstrating their key roles in this bacterium. Some medicines, such as Orlistat, have been reported to be able to prevent the growth of *A. baumannii* by inhibiting LipA [36], while blocking the function of GspD or the pathways of Sec and Tat can contribute to the inhibition of virulence mediated by the T2SS [117]. Conserved sequences of VgrG were identified to be antigenic in various strains of *A. baumannii*; thus, it could be used to develop multivalent vaccines [118,119]. In addition, the T5SS protein, Ata, has been reported to be a potential candidate vaccine by many researchers because of its outstanding protection effect against the lethal challenges of various *A. baumannii* strains [120,121,122]. Additionally, further investigations on more effectors of the T2SS and T6SS, as well as other less reported secretion systems, may offer further opportunities to control the infections caused by drug-resistant *A. baumannii*.

## Data Availability

Not applicable.
